# HIV drug resistance in a community‐randomized trial of universal testing and treatment: HPTN 071 (PopART)

**DOI:** 10.1002/jia2.25941

**Published:** 2022-07-01

**Authors:** Jessica M. Fogel, Ethan A. Wilson, Estelle Piwowar‐Manning, Autumn Breaud, William Clarke, Christos Petropoulos, Ayana Moore, Christophe Fraser, Barry Kosloff, Kwame Shanaube, Gert van Zyl, Michelle Scheepers, Sian Floyd, Peter Bock, Helen Ayles, Sarah Fidler, Richard Hayes, Deborah Donnell, Susan H. Eshleman, for the HPTN 071 (PopART) Study Team

**Affiliations:** ^1^ Department of Pathology Johns Hopkins University School of Medicine Baltimore Maryland USA; ^2^ Fred Hutchinson Cancer Research Center Seattle Washington USA; ^3^ Monogram Biosciences San Francisco California USA; ^4^ FHI 360 Durham North Carolina USA; ^5^ The Big Data Institute and Wellcome Centre for Human Genetics, Nuffield Department of Medicine University of Oxford Oxford UK; ^6^ Zambart Lusaka Zambia; ^7^ Department of Clinical Research London School of Hygiene and Tropical Medicine London UK; ^8^ Division of Medical Virology Stellenbosch University Cape Town South Africa; ^9^ Desmond Tutu TB Center, Department of Paediatrics and Child Health Stellenbosch University Western Cape South Africa; ^10^ Department of Infectious Disease Epidemiology London School of Hygiene and Tropical Medicine London UK; ^11^ Department of Infectious Disease HIV Clinical Trials Unit, Imperial College London London UK

**Keywords:** Africa, ARV, clinical trials, drug resistance, HIV prevention, transmitted drug resistance

## Abstract

**Introduction:**

Universal HIV testing and treatment (UTT) has individual and public health benefits. HPTN 071 (PopART), a community‐randomized trial in Zambia and South Africa, demonstrated that UTT decreased HIV incidence. This endpoint was assessed in a cohort of >48,000 randomly selected adults in the study communities. We evaluated the impact of UTT on HIV drug resistance in this cohort and compared other resistance‐related outcomes in participants with recent versus non‐recent HIV infection.

**Methods:**

Two years after the start of HPTN 071 (2016–2017), 6259 participants were HIV positive and 1902 were viremic (viral load >400 copies/ml). HIV genotyping and antiretroviral (ARV) drug testing were performed for viremic participants in three groups: seroconverters (infected <1 year), non‐seroconverters (infected >1 year, random subset) and participants with unknown duration of infection (random subset). A two‐stage cluster‐based approach was used to assess the impact of the study intervention on drug resistance. Treatment failure was defined as being viremic with ARV drugs detected. Participants were classified as ARV naïve based on self‐report and ARV drug testing.

**Results:**

Genotyping results were obtained for 758 participants (143 seroconverters; 534 non‐seroconverters; and 81 unknown duration of infection). The estimated prevalence of resistance in the study communities was 37% for all viremic persons and 11% for all HIV‐positive persons. There was no association between UTT and drug resistance. Resistance was detected in 14.0% of seroconverters and 40.8% of non‐seroconverters (non‐nucleoside reverse transcriptase inhibitor resistance: 14.0% and 39.9%; nucleoside/nucleotide reverse transcriptase inhibitor resistance: 0.7% and 15.5%; protease inhibitor resistance: 0% and 1.9%; multi‐class resistance: 0.7% and 16.1%, respectively). ARV drugs were detected in 2/139 (1.4%) of seroconverters and 94/534 (17.6%) of non‐seroconverters tested. These participants were classified as failing ART; 88 (93.6%) of the non‐seroconverters failing ART had resistance. Mutations used for surveillance of transmitted drug resistance were detected in 10.5% of seroconverters and 15.1% of non‐seroconverters who were ARV naive.

**Conclusions:**

UTT was not associated with an increase in drug resistance in this cohort. Higher rates of drug resistance and multi‐class resistance were observed in non‐seroconverters compared to seroconverters.

## 1 INTRODUCTION

In 2011, the HIV Prevention Trials Network (HPTN) 052 trial demonstrated that early initiation of antiretroviral therapy (ART) is highly effective for preventing sexual transmission of HIV [1]. Early ART initiation also had health benefits for people living with HIV (PLHIV) [2, 3]. These findings supported changes in global guidelines for ART that included increased HIV testing with ART initiated at any CD4 cell count (universal testing and treatment, UTT) [4]. Use of antiretroviral (ARV) drugs for HIV prevention and treatment can be compromised by HIV drug resistance. Increasing rates of drug resistance have been observed in low‐ and middle‐income countries [5, 6, 7]. Factors potentially contributing to the rise in drug resistance in these settings include increased use of ARV drugs, poor ART adherence, limited access to viral load monitoring and drug resistance testing, unreliable drug supply chains, use of less potent ART regimens, prior ARV drug exposure and transmission of drug‐resistant virus [5, 7]. Few studies have evaluated whether provision of UTT is associated with an increase in drug resistance [8, 9, 10]. Increased uptake of ART in the setting of UTT could potentially increase the prevalence of drug resistance, especially in settings with limited access to routine viral load monitoring and drug resistance testing.

In this report, we assessed HIV drug resistance in a large, community‐randomized trial that evaluated the impact of UTT on HIV incidence, HPTN 071 (PopART). This trial was conducted in 21 communities in South Africa and Zambia [11]. Communities were grouped in triplets and randomized to one of three study arms. Arm A communities received a combination prevention package that included UTT (door‐to‐door HIV testing and counselling with immediate ART for all PLHIV). Arm B communities received the same combination prevention package with ART provided according to local guidelines. Arm C communities received HIV testing services and ART according to local guidelines. During the course of the trial, local guidelines for ART changed and the criteria for ART initiation in Arms B and C were revised (from ART initiation at CD4 <350 cells/mm^3^, to CD4 <500 cells/mm^3^, to universal ART). In the latter portion of the study, the intervention in Arm B was similar to the intervention in Arm A.

In HPTN 071, HIV testing was performed at baseline and in three annual surveys. The primary outcome of HIV incidence in HPTN 071 was measured after full implementation of the study intervention (between the 1‐ and 3‐year surveys). The adjusted rate ratio for HIV incidence was significantly lower for Arm B compared to Arm C, but was not significantly different for Arm A compared to Arm C [11]. The secondary outcome of community viral load was measured 2 years after study implementation. At the 2‐year survey, the mean community proportion of PLHIV who were virally suppressed (viral load <400 copies/ml) was 71.9% in Arm A, 67.5% in Arm B and 60.2% in Arm C [11]. The percentage of seroconverters who were virally suppressed at their first HIV‐positive visit increased during the course of the study (25% at 1 year, 30% at 2 years and 33% at 3 years) [12], suggesting an increase in access to early ART over time.

The main focus of this report is to evaluate whether the HPTN 071 study intervention (UTT) was associated with an increase in HIV drug resistance. Additional assessments were performed to compare resistance‐related outcomes in persons with recent infection (infected <1 year) and non‐recent infection (infected >1 year). These assessments included the prevalence and type of drug resistance detected; association of drug resistance with demographic factors and ARV drug use; and transmitted drug resistance.

## 2 METHODS

### 2.1 Samples used for analysis

In HPTN 071, key study outcomes were assessed in a population cohort that included a random sample of >48,000 adults aged 18–44 (one per household), reflecting the general adult population. Participants were recruited over the first 2 years of the study and were followed for up to 3 years at annual study visits (at baseline and at the 1‐, 2‐ and 3‐year surveys; File S1); HIV testing was performed at each annual visit [12].

Samples analysed in this report were obtained in the 2‐year survey from PLHIV in the population cohort (collection dates: August 2016–July 2017). This survey year was selected for analysis rather than the 3‐year survey since universal ART was implemented as standard‐of‐care in all study communities in the last year of the trial (File S1); this would make it more difficult to detect an effect of the study intervention on drug resistance in the 3‐year survey. HIV genotyping and ARV testing were performed for PLHIV who had viral loads >400 copies/ml. This study describes outcomes in three participant groups: seroconverters (HIV negative at the 1‐year survey and HIV positive at the year‐2 survey; infected <1 year), non‐seroconverters (HIV positive at the 1‐ and 2‐year surveys; infected >1 year) and participants with unknown duration of infection (HIV status not determined at the 1‐year survey). Due to the large number of PLHIV in the 2‐year survey, HIV drug resistance was analysed for all seroconverters and a randomly selected subset of other PLHIV (21–30 participants per community).

### 2.2 Laboratory assays

Viral load testing was performed using the Abbott RealTime HIV‐1 Viral Load assay (Abbott Molecular, Abbott Park, IL; validated dilution method, limit of quantification: 400 copies/ml). HIV genotyping was performed using the GenoSure MG assay (Monogram Biosciences, South San Francisco, CA). This assay evaluates the susceptibility to non‐nucleoside reverse transcriptase inhibitors (NNRTIs), nucleoside/nucleotide reverse transcriptase inhibitors (NRTIs) and protease inhibitors (PIs). Drug susceptibility is reported as sensitive, resistance possible or resistant. In this study, samples were classified as “resistant” if the testing laboratory reported resistance to one or more ARV drug. The GenoSure MG assay also provides the HIV subtype for each sample. Major drug resistance mutations (DRMs) were identified using the IAS Drug Resistance 2019 report [13]. Mutations recommended for surveillance of transmitted drug resistance [14] were used to evaluate transmitted drug resistance in ARV‐naïve participants (no history of ARV drug exposure and no ARV drugs detected in their sample). ARV drug testing was performed using a qualitative assay based on high‐resolution accurate mass spectroscopy [15, 16]. This assay detects 22 ARV drugs (three NNRTIs, six NRTIs, nine PIs, three integrase inhibitors and one CCR5‐antagonist) with a limit of detection of 5, 20, 50 or 150 ng/ml, depending on the drug. ART failure was defined as having a viral load >400 copies/ml with ARV drugs detected.

### 2.3 Statistical analysis

Chi‐square tests were used to assess the associations between risk factors and drug resistance (SAS v9.4). Differences in the prevalence of drug resistance between study arms were assessed using a two‐stage analysis approach for matched cluster‐randomized trials [17]. In the trial design, the matched triplets largely accounted for regional and demographic differences between communities; triplets were matched on HIV prevalence and geographic location. We assessed the impact of the HPTN 071 study intervention on resistance in all PLHIV and in PLHIV who had a viral load >400 copies/ml. For this analysis, we conducted a two‐way ANOVA on triplet and study arm of the log ratio of the observed and expected proportions of included participants in each community who had a viral load >400 copies/ml and had drug resistance. The observed proportions with this outcome (viral load >400 copies/ml with drug resistance) were estimated in each study community using survey logistic regression, based on the known probabilities of being selected for resistance testing. The expected proportions were modelled using survey logisitic regression adjusted for triplet, age category and sex, allowing for different prevalence of resistance by triplet but no intervention effect. This analysis included all three participant groups (seroconverters, non‐seroconverters and participants with unknown duration of infection) to reflect all viremic PLHIV included in the 2‐year survey. A description of the methods used for this analysis is provided in File S2.

### 2.4 Ethical considerations

Written informed consent was obtained from all population cohort participants. Ethical approval for the HPTN 071 trial was granted by ethics committees at the London School of Hygiene & Tropical Medicine, University of Zambia and Stellenbosch University.

## 3 RESULTS

### 3.1 Study cohort

At the 2‐year survey, 6259 participants in the population cohort tested positive for HIV infection, including 225 seroconverters. Among the seroconverters, 161 (71.6%) had a viral load >400 copies/ml. Genotyping results were obtained for 143 (88.8%) of the 161 samples. The remaining 6034 participants included 5014 non‐seroconverters and 1020 participants with unknown duration of infection; 1741 of those participants had a viral load >400 copies/ml (1374 non‐seroconverters and 367 persons with unknown duration of infection). A random subset of those participants was selected for resistance testing (566 non‐seroconverters and 90 participants with unknown duration of infection; this subset included ∼38% of the non‐seroconverters and participants with unknown infection duration in the study communities). Genotyping results were obtained for 534 (94.3%) of non‐seroconverter samples and 81 (90.0%) of samples from those with unknown infection duration (File S3). Most (95.9%) of the 758 participants with genotyping results had subtype C HIV infection.

### 3.2 Prevalence of drug resistance

We first evaluated the overall prevalence of drug resistance in the study communities. This assessment included data from all 758 participants with genotyping results (all three participant groups). Since drug resistance was only assessed in a random subset of participants in two groups (non‐seroconverters and persons with unknown duration of infection), we first evaluated whether participants with genotyping results were representative of the study communities by comparing the demographic characteristics of participants with genotyping results to the larger group of all eligible PLHIV who had viral loads >400 copies/ml. The demographic characteristics were similar in these two groups, with one exception: the group with genotyping results included a lower proportion of participants enrolled at the 2‐year survey (8.2% among those with genotyping results and 16.7% among all eligible participants, data not shown). HIV genotyping results were then weighted for sampling to estimate the prevalence of resistance in the study communities. The overall estimated prevalence of drug resistance in the study communities was 11% among all PLHIV and 37% among all viremic persons.

### 3.3 Impact of the study intervention

We next evaluated the relationship between HIV drug resistance and the study intervention (UTT). This assessment included all 758 participants with genotyping results (all three participant groups). We did not find an association between drug resistance and UTT among all PLHIV or among PLHIV who were viremic (viral load>400 copies/ml) (Table 1). Among all PLHIV, the relative proportion of participants with resistance was 0.95 (95% confidence interval [CI]: 0.68, 1.31, *p* = 0.72) for Arm A versus C and 1.05 (95% CI: 0.76, 1.45, *p* = 0.76) for Arm B versus C. Among all PLHIV who were viremic, the relative proportion of participants with resistance was 1.18 (95% CI: 0.96–1.44, *p* = 0.11) for Arm A versus C and 1.15 (95% CI: 0.93–1.40, *p* = 0.17) for Arm B versus C. Additional assessments were performed to evaluate the resistance‐related outcomes in the 143 seroconverters and 534 non‐seroconverters who had genotyping results (see below). Participants with unknown duration of infection were not included in those assessments.

**Table 1:** Impact of the HPTN 071 study intervention on HIV drug resistance

		Prevalence of HIV drug resistance at the 2‐year survey		
Group analysed	Study arm	Unadjusted observed (SE)^a^	Adjusted ratio^b^ (95% CI)	*p* value
All HIV positive	A	12% (0.044)		
	B	11% (0.040)		
	C	10% (0.036)		
	Overall	11% (0.012)		
	A versus C		0.95 (0.68, 1.31)	0.72
	B versus C		1.05 (0.76, 1.45)	0.76
All HIV positive with viral load >400 copies/ml	A	42% (0.022)		
	B	39% (0.37)		
	C	33% (0.018)		
	Overall	37% (0.017)		
	A versus C		1.18 (0.96, 1.44)	0.11
	B versus C		1.15 (0.93, 1.40)	0.17

Note: The table shows the results for analysis of HIV drug resistance by study arm in the HPTN 071 trial. Additional information on the statistical methods used for this analysis is provided in File S2.

Abbreviations: CI, confidence interval; SE, standard error.

^a^ Prevalence estimate accounting for sampling weights.

^b^ Adjusted for age, sex, age/sex interaction and community triplet.

### 3.4 Drug resistance in seroconverters versus non‐seroconverters

The demographic characteristics of the seroconverters and non‐seroconverters with genotyping results are shown in File S4. Seroconverters were more likely to be younger (18–24 years) compared to non‐seroconverters (44.1% vs. 16.5%, *p* < 0.001); enrolment country and sex were not significantly different in these two groups (Table 2).

**Table 2:** Comparison of drug resistance, antiretroviral drug use and transmitted drug resistance among seroconverters and non‐seroconverters

	Seroconverters	Non‐seroconverters	*p*‐value
# HIV positive	225	5014	
# viremic (VL>400 copies/ml)	161 (71.6%)	1374 (27.4%)	
# selected for analysis	161 (100%)	566 (41.2%)	
HIV genotyping results obtained^a^	143/161 (88.8%)	534/566 (94.3%)	
# (%) with resistance	20/143 (14.0%)	218/534 (40.8%)	**<0.001**
# (%) with multi‐class resistance	1/143 (0.7%)	86/534 (16.1%)	**<0.001**
# (%) tested for ARV drugs^b^	139/143 (97.2%)	534/534 (100%)	
# (%) failing ART^c^	2/139 (1.4%)	94/534 (17.6%)	**<0.001**
# (%) with resistance among those failing ART^c^	0/2 (0%)	88/94 (93.6%)	**0.006**
# (%) ARV naïve^d^	133/136 (97.8%)	325/521 (62.4%)	**<0.001**
# (%) with transmitted drug resistance^e^	14/133 (10.5%)	49/325 (15.1%)	0.20

Bold indicates *p* < 0.05.

^a^ Among the seroconverters: 13 failed testing, one did not have a sample available for resistance testing and four were excluded who had acute HIV infection; among the non‐seroconverters: 32 failed testing.

^b^ Four seroconverters did not have samples available for antiretroviral (ARV) drug testing.

^c^ ART failure was defined as having a viral load >400 copies/ml with ARV drugs detected.

^d^ Participants were classified as ARV naïve based on self‐report and ARV drug testing. Data for this assessment were available for 136 (95.1%) of 143 seroconverters and 521 (97.6%) of the 534 non‐seroconverters.

^e^ Participants were classified as having transmitted drug resistance if they were ARV naïve and had mutations detected that are used for surveillance of transmitted resistance.

**Figure 1:**
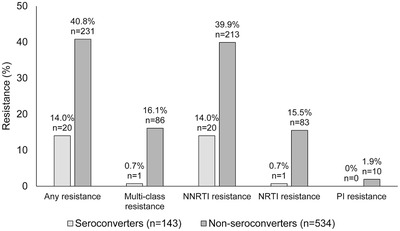
Prevalence of HIV drug resistance. Note: The figure shows the prevalence of drug resistance among seroconverters and non‐seroconverters at the 2‐year survey. Overall resistance (any resistance), multi‐class resistance (MCR) and resistance to individual drug classes are shown. Abbreviations: NNRTI, non‐nucleoside reverse transcriptase inhibitor; NRTI, nucleoside/nucleotide reverse transcriptase inhibitor; PI, protease inhibitor.

Among the 143 seroconverters with genotyping results, 20 (14.0%) had NNRTI resistance, one (0.7%) had NRTI resistance and none had PI resistance. Among the 534 non‐seroconverters with genotyping results, 213 (39.9%) had NNRTI resistance, 83 (15.5%) had NRTI resistance and 10 (1.9%) had PI resistance. Multi‐class resistance was observed in 1 (0.7%) of the 143 seroconverters (NNRTI+NRTI resistance) and 86 (16.1%) of the 534 non‐seroconverters (81 had NNRTI+NRTI resistance, three had NNRTI+PI resistance and two had NNRTI+NRTI+PI resistance) (Figure 1 and Table 2).

**Figure 2:**
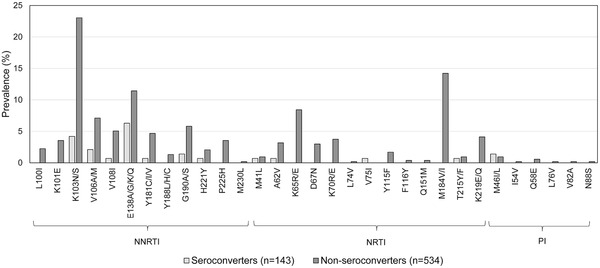
Major drug resistance mutations detected. Note: The figure shows the major drug resistance mutations (DRMs) detected among seroconverters and non‐seroconverters at the 2‐year survey. Major DRMs that were detected in >5% of seroconverters are as follows: NNRTI mutations: E138A/K (9 [6.3%]; E138A only: 8 [5.6%]). Major DRMs that were detected in >5% of non‐seroconverters are as follows: NNRTI mutations: K103N/S (123 [23.0%], E138A/G/K/Q (61 [11.4%]; E138A only: 49 [9.2%]), V106M/A (38 [7.1%]), G190A/S (31 [5.8%]), V108I (27 [5.1%]); NRTI mutations: M184V/I (76 [14.2%]) and K65R/E (45 [8.4%]; K65R only: 44 [8.2%]). Abbreviations: NNRTI, non‐nucleoside reverse transcriptase inhibitor; NRTI, nucleoside/nucleotide reverse transcriptase inhibitor.

The major DRMs detected are shown in Figure 2. The most common DRMs detected in the seroconverters were the NNRTI DRMs E138A/K (6.3%) and K103N (4.2%). The most common DRMs detected in the non‐seroconverter group were the NNRTI DRMs K103N (23.0%) and E138A/G/K/Q (11.4%), and the NRTI DRMs M184V/I (14.2%) and K65R/E (8.4%).

Prevalence of drug resistance and multi‐class resistance was significantly lower among seroconverters compared to non‐seroconverters (*p* < 0.001 for both, Table 2). There was no association of drug resistance with country, age or sex in either group (Table 3).

**Table 3:** Factors associated with HIV drug resistance

		Seroconverters *N* = 143				Non‐seroconverters *N* = 534			
Variable		*N*	*N* (%) with resistance	*p* value	OR (95% CI)	*N*	*N* (%) with resistance	*p* value	OR (95%CI)
Country	South Africa	55	8 (14.5%)	0.88	1	217	96 (44.2%)	0.18	1
	Zambia	88	12 (13.6%)		0.93 (0.35–2.44)	317	122 (38.5%)		0.79 (0.55–1.12)
Age	18–24 years	63	7 (11.1%)	0.38	1	88	30 (34.1%)	0.16	1
	25+ years	80	13 (16.3%)		1.55 (0.58–4.16)	446	188 (42.2%)		1.41 (0.87–2.27)
Sex	Male	24	4 (16.7%)	0.68	1.29 (0.39–4.26)	104	39 (37.5%)	0.44	0.84 (0.54–1.31)
	Female	119	16 (13.4%)		1	430	179 (41.6%)		1
ARV drugs detected^a^	Yes	2	0 (0%)	1.0	–	94	88 (93.6%)	**<0.001**	**35.0 (14.9–82.0)**
	No	137	19 (13.9%)			440	130 (29.5%)		

Bold indicates *p* < 0.05.

Note: The table shows demographic characteristics, ARV drug testing results and drug resistance results for seroconverters and non‐seroconverters. There were insufficient data to evaluate the association of HIV drug resistance with specific antiretroviral treatment (ART) regimens.

Abbreviations: ARV, antiretroviral; CI, confidence interval; *N*, number; OR, odds ratio.

^a^ Four seroconverters did not have results for ARV drug testing.

### 3.5 Association of ARV drug use and drug resistance

ARV drug testing was performed for participants with genotyping results (all with viral loads >400 copies/ml). ARV drugs were detected in 2 (1.4%) of 139 samples from seroconverters (four samples were not available for testing) and 94 (17.6%) of 534 samples from non‐seroconverters (Table 2); these participants were considered to be failing ART.

The most common drug‐regimen detected was one NNRTI with one or two NRTIs (seroconverters: 2/2; non‐seroconverters: 72/94). The most common combination of drugs detected in South Africa was efavirenz/lamivudine/tenofovir and the most common combination of drugs detected in Zambia was efavirenz/emtricitabine/tenofovir (File S5). Neither of the seroconverters failing ART had drug resistance. In contrast, almost all (88/94 [93.6%]) of the non‐seroconverters failing ART had drug resistance (Table 2). Resistance was associated with detection of ARV drugs among non‐seroconverters (*p* < 0.001, Table 3).

### 3.6 Transmitted drug resistance

We next analysed the frequency of transmitted drug resistance among ARV‐naïve participants. Participants were classified as ARV naïve if (1) they reported no past or current ART and no use of ARV drugs for prevention of mother‐to‐child transmission of HIV at their last pregnancy, and (2) if they had no ARV drugs detected. Using these criteria, 133 seroconverters and 325 non‐seroconverters were classified as ARV naïve. This analysis was limited to mutations recommended for surveillance of transmitted drug resistance [14]; this list does not include the mutations E138A/K, which occur as natural polymorphisms in non‐subtype B [13].

Fourteen (10.5%) of the 133 ARV‐naïve seroconverters had mutations on the surveillance list and were classified as having transmitted drug resistance (Table 2 and File S6). Ten of these participants were women and 11 were >25 years of age. Nine of the 14 participants had one NNRTI resistance mutation only and one had multi‐class resistance.

Forty‐nine (15.1%) of the 325 ARV naive non‐seroconverters had mutations on the surveillance list and were classified as having transmitted drug resistance (Table 2 and File S6). Thirty‐two of these participants had one NNRTI resistance mutation only. The other 17 participants had the following patterns of surveillance mutations: two NNRTI resistance mutations (*n* = 4); a single NRTI resistance mutation (*n* = 1); a single PI resistance mutation (*n* = 8); and multi‐class resistance (NNRTI resistance with NRTI or PI resistance, *n* = 4).

## 4 DISCUSSION

This study evaluated HIV drug resistance among participants in HPTN 071 2 years after study implementation. The overall estimated prevalence of drug resistance was 11% among all PLHIV and 37% among all viremic PLHIV in the study communities. This is higher than estimates from a 2017 national household survey in South Africa that reported a prevalence of resistance of 27% among viremic PLHIV of all ages [10]. However, it is difficult to compare results from these studies, since different methods were used for resistance testing and analysis. There was no evidence that implementation of a prevention package that included UTT increased drug resistance compared to standard‐of‐care services. In a cluster‐based analysis, there was no significant difference in the prevalence of drug resistance between either of the study intervention arms (Arm A or Arm B) and the standard‐of‐care arm (Arm C), among all PLHIV or among the subset of PLHIV who had HIV viral loads >400 copies/ml.

Additional assessments were performed for seroconverters and non‐seroconverters who had genotyping results. Drug resistance was detected in 14.0% of seroconverters and 40.8% of non‐seroconverters; 16.1% of the non‐seroconverters had multi‐class resistance. There was no significant association of drug resistance with country, age or sex in either group. Drug resistance was highly associated with detection of ARV drugs in non‐seroconverters, but not in seroconverters. In this study, only two (1.4%) of the seroconverters with genotyping results had ARV drugs detected. This suggests that most of the viremic seroconverters were not on ART and that ART failure was rare in this group. In contrast, 17.6% of non‐seroconverters with genotyping results had ARV drugs detected. This corresponds to ∼5% of the non‐seroconverters in the study communities. Nearly, all (93.6%) of the participants failing ART had resistance.

NNRTI and NRTI resistance were frequent among non‐seroconverters (39.9% and 15.5%, respectively); PI resistance was uncommon (detected in 1.9%). This was consistent with the low frequency of PI use detected among participants in this group. The most common NRTI mutations detected in non‐seroconverters were M184V/I (14.2%) and K65R/E (8.4%), which are associated with resistance to tenofovir and emtricitabine. This suggests that the use of tenofovir disoproxil fumarate/emtricitabine for pre‐exposure prophylaxis (PrEP) in this population could be compromised by drug resistance.

This study assessed resistance using samples collected in 2016 and 2017. Integrase strand transfer inhibitor (INSTI) resistance was not evaluated in this study, since INSTIs were not in widespread use in Zambia and South Africa at that time; none of the samples analysed had INSTI drugs detected. In 2019, the World Health Organization guidelines were updated to include the use of dolutegravir‐based regimens for first‐line ART for all adolescents and adults [18]. A long‐acting injectable form of the INSTI cabotegravir was recently approved by the U.S. Food and Drug Administration for maintenance of viral suppression among those on ART and for HIV PrEP [19, 20]. Going forward, it will be important to include INSTIs in resistance surveillance studies in these regions.

The prevalence of transmitted drug resistance in this study was 10.5% among seroconverters and 15.1% among non‐seroconverters. In a 2014 meta‐analysis of the global burden of transmitted drug resistance, a trend of increasing prevalence was observed in low‐ and middle‐income countries (for the period 2009–2013, transmitted drug resistance prevalence was ∼8% among men who have sex with men, ∼4% among heterosexuals and ∼5% among persons who inject drugs); in sub‐Saharan Africa, prevalence was ∼2% [21]. It is difficult to compare the rates of transmitted drug resistance across studies, since different participant groups are evaluated (e.g. those with recent diagnosis or recent/incident infection) and different measures are used to identify those who are likely to be ARV drug naïve (e.g. drug testing, self‐report of ARV drug use for ART or other indications). Prior studies demonstrate that participants in research studies may not report prior knowledge of HIV infection or ARV drug use [22, 23, 24]. In this study, self‐report and ARV drug testing was used to identify ARV‐naïve participants. The higher rate of transmitted drug resistance that we observed in non‐seroconverters may reflect unreported past use of ARV drugs for ART or other reasons.

This study has some limitations. In HPTN 071, surveys were conducted annually. Therefore, it was not possible to assess resistance close to the time of infection. Second, because of the large size of the trial, we were only able to analyse resistance in a subset of participants in two of the groups studied (non‐seroconverters and persons with unknown duration of infection). This is not likely to have impacted the findings of this study, since the subgroup was randomly selected and had similar demographic characteristics to those in the larger cohort. Third, local guidelines for ART initiation changed during the conduct of the trial and the timeline for these changes was different in South Africa and Zambia. The analysis in this report was also performed after 2 years of the study intervention (rather than 3), since universal ART was recommended in all study communities in the last year of the trial. These factors may have made it more difficult to detect an impact of UTT on drug resistance, but were not likely to have impacted the other assessments in this report. Fourth, some participants classified as failing ART may have initiated ART shortly before sample collection and not yet achieved viral suppression; this would tend to overestimate the frequency of ART failure. Fifth, some participants classified as ARV naïve may have had prior drug exposure (e.g. if this was not reported to study staff, or if drugs were not detected because the participants were not taking ARV drugs in the weeks prior to sample collection). Also, participants were not asked about the use of ARV drugs for other reasons. Sixth, despite the large size of the HPTN 071 population cohort, the number of participants with transmitted drug resistance was small. This limited our ability to identify factors associated with transmitted drug resistance. Last, the number of seroconverters in the study was too small for meaningful analysis of the impact of UTT on resistance in this group.

## 5 CONCLUSIONS

In summary, we found high rates of HIV drug resistance among viremic participants in the HPTN 071 trial, especially among those who acquired HIV >1 year prior to testing and among those who were actively using ARV drugs. The UTT intervention was not associated with an increase in drug resistance. Increased support for ART adherence, resistance monitoring and use of drugs with high genetic barriers to resistance may help reduce resistance in these settings.

## COMPETING INTERESTS

None of the authors have any competing interests.

## AUTHORS’ CONTRIBUTIONS

All authors participated in the study, contributed to manuscript preparation and reviewed the manuscript. Additional roles are shown as: JMF designed the study, analysed data and drafted the manuscript. EAW was HPTN 071 Statistical Research Associate. EPM was HPTN 071 QAQC Coordinator.

AB performed antiretroviral drug testing. WC provided scientific oversight for antiretroviral drug testing. CP provided scientific oversight for resistance testing.

AM was HPTN 071 Study Coordinator. CF provided input on population‐level drug resistance in HPTN 071. BK and KS provided laboratory support for HPTN 071 in Zambia. GvZ and MS provided laboratory support for HPTN 071 in South Africa. SF was HPTN 071 Senior Statistician (LSHTM). PB was HPTN 071 South African Site Co‐PI. HA was HPTN 071 Zambian Site PI. SF was HPTN 071 Protocol Co‐Chair. RH was HPTN 071 Protocol Chair. DD was HPTN 071 Protocol Statistician. SHE was HPTN 071 Virologist; designed the study, analysed data and drafted the manuscript.

## FUNDING

This work was supported by the HIV Prevention Trials Network (HPTN) sponsored by the National Institute of Allergy and Infectious Diseases (NIAID) under Cooperative Agreements UM1‐AI068619, UM1‐AI068617 and UM1‐AI068613, with funding from the U.S. President's Emergency Plan for AIDS Relief (PEPFAR). Additional funding was provided by the International Initiative for Impact Evaluation (3ie) with support from the Bill & Melinda Gates Foundation, as well as by NIAID, the National Institute on Drug Abuse (NIDA) and the National Institute of Mental Health (NIMH), all part of NIH.

## DISCLAIMER

The content is solely the responsibility of the authors and does not necessarily represent the official views of the NIAID, NIMH, NIDA, PEPFAR, 3ie or the Bill & Melinda Gates Foundation. RH and SF also received support from the UK Medical Research Council (MRC) and the UK Department for International Development (DFID) under the MRC/DFID Concordat agreement, which is also part of the EDCTP2 programme supported by the European Union (MR/R010161/1). Additional support was provided by the Division of Intramural Research, NIAID.

## Supporting information


**File S1**. Overview of the HPTN 071 (PopART) trial.
**File S2**. Statistical methods used to analyse the effect of the HPTN 071 study intervention on HIV drug resistance.
**File S3**. Study cohort.
**File S4**. Demographic characteristics of seroconverters and non‐seroconverters.
**File S5**. Antiretroviral drug testing.
**File S6**. Characteristics of participants with transmitted drug resistance.

**File S7**. HIV subtypes and mutations detected.

## Data Availability

The data used in the manuscript are available in File S7. This file includes the HIV subtype and a list of mutations and polymorphisms in each sample identified at the testing laboratory.
