# Supplementary material for: HIV drug resistance in a community‐randomized trial of universal testing and treatment: HPTN 071 (PopART)
Source: J Int AIDS Soc. 2022 Jul 1;25(7):e25941. doi: 10.1002/jia2.25941 (PMC9248006; doi:10.1002/jia2.25941)
Supplement: Supplementary file 1 — File S1. Overview of the HPTN 071 (PopART) trial. File S2. Statistical methods used to analyse the effect of the HPTN 071 study intervention on HIV drug resistance. File S3. Study cohort. File S4. Demographic characteristics of seroconverters and non‐seroconverters. File S5. Antiretroviral drug testing. File S6. Characteristics of participants with transmitted drug resistance. [file JIA2-25-e25941-s002.docx]

**SUPPLEMENTAL FILES**

| Supplemental File 1 | Overview of the HPTN 071 (PopART) trial |
| --- | --- |
| Supplemental File 2 | Statistical methods used to analyze the effect of the HPTN 071 study intervention on HIV drug resistance |
| Supplemental File 3 | Study cohort |
| Supplemental File 4 | Demographic characteristics of seroconverters and non-seroconverters |
| Supplemental File 5 | Antiretroviral drug testing |
| Supplemental File 6 | Characteristics of participants with transmitted drug resistance |

**Supplemental File 1**. Overview of the HPTN 071 (PopART) trial.

HPTN 071 (PopART) was conducted in 21 communities in Zambia and South Africa (total population approximately 1 million). Detailed descriptions of study procedures and outcomes are described in the primary study report (Hayes et al, New Engl J Medicine. 2019; 381:207-218). Communities were grouped in triplets (matched by location and HIV prevalence) and were randomly assigned to one of three study arms. Arm A communities received a combination prevention intervention that included universal antiretroviral therapy (ART, at any CD4 cell count). Arm B communities received the combination prevention intervention with ART provided according to local guidelines. Arm C communities received standard care. The combination prevention intervention included home visits by community workers who performed HIV testing and provided support for linkage to care and adherence to ART. A Population Cohort of randomly-selected adults (>48,000; aged 18-44) from households in each community were enrolled to evaluate the primary trial outcome: the effect of the intervention on population-level HIV incidence. Home visits were conducted in four annual surveys, referred to as PC0 (baseline), PC12 (1-year survey), PC24 (2-year survey), and PC36 (3-year survey).

The figure shows the timeline for each survey conducted in the Population Cohort and the guidelines for ART initiation in each country over the course of the HPTN 071 trial. ART was provided through local facilities under the Ministry of Health in Zambia and the Department of Health in South Africa. At the start of the trial, ART regimens recommended in the study communities were efavirenz/lamivudine/tenofovir for first-line ART and lopinavir/ritonavir/zidovudine/lamivudine for second-line ART. Local guidelines for ART initiation changed during the conduct of the HPTN 071 study; universal ART was provided in all study communities near the start of the PC24 survey. Samples analyzed in this report were collected at the 2-year survey.


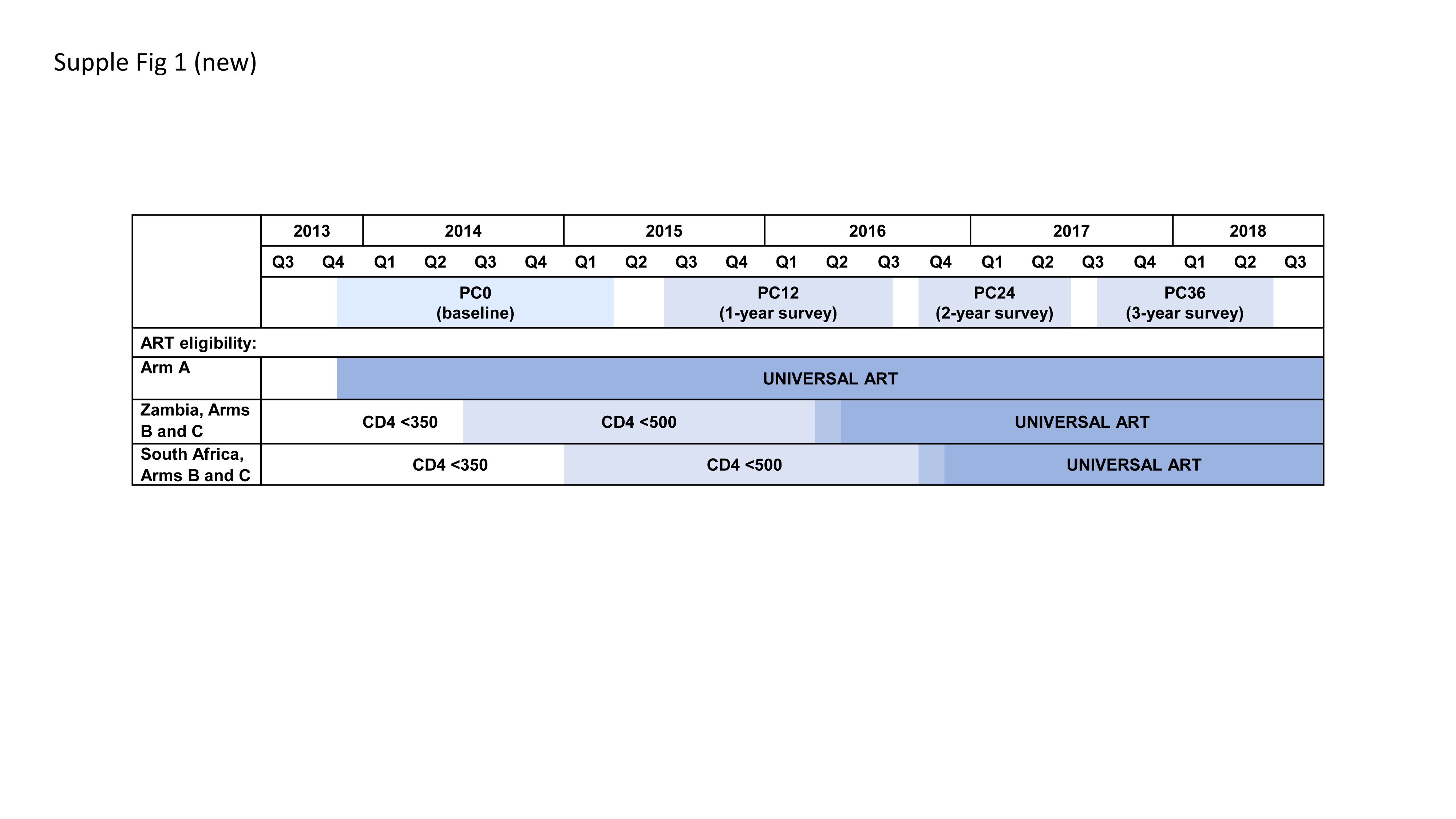


Abbreviations: Q: calendar year quarter; PC: Population Cohort: ART: antiretroviral therapy; CD4: CD4 cell count criteria for ART initiation.

**Supplemental File 2**. Statistical methods used to analyze the effect of the HPTN 071 study intervention on HIV drug resistance.

HPTN 071 was a triplet-matched, cluster-randomized trial. The analysis used in this report followed the two-stage statistical analysis approach that was used for analysis of the primary and secondary outcomes of the trial. The methods were modified as noted below because resistance testing was only performed for a random subset of participants in some subgroups. The outcome of the analysis was HIV infection with a viral load >400 copies/mL with HIV drug resistance. The same analysis was conducted using two cohorts: one that included all persons living with HIV (PLHIV), and one that was restricted to PLHIV who had viral loads >400 copies/mL.

In the first stage of the analysis, we estimated the expected number of individuals with the outcome in each community under the null hypothesis of no intervention effect, allowing for different proportions with the outcome in each triplet, and adjusted for age category and sex. We predicted the expected number ($E_{ij}$; ith community in the jth triplet) using survey logistic regression, with covariates triplet, age category and sex, and sampling weights of the probability of being selected. We estimated the observed number of participants with the outcome in each community ($O_{ij}$) using survey logistic regression with community as the only predictor, with the same sampling weights. Unadjusted observed prevalence by arm and overall are predictions from the survey logistic regression.

In the second stage of the analysis, we computed the log ratio-residual for each community = $\log\left( O_{\mathrm{ij}}/E_{\mathrm{ij}} \right).$ A two-way ANOVA of triplet and arm was used to assess intervention effect in the log ratio-residuals of the 21 study communities. P-values and 95% confidence intervals were based on Student’s t-distribution with 12 degrees of freedom. Analyses were carried out in R version 4.0.2 (2020-06-22), including requisite packages ‘tidyverse’ and ‘survey’.

**Supplemental File 3**. Study cohort.





Legend for Figure.

The figure shows the total number of HIV-positive participants at the 2-year survey, the number with a viral load (VL) >400 copies/mL, the number selected for HIV genotyping, and the number with genotyping results. Samples without genotyping results failed testing.

^a^ Five samples were not tested (one had insufficient plasma for testing and four were from participants with acute HIV infection

^b^ A random subset of participants in these subgroups was selected for testing (21 to 30 per community).

**Supplemental File 4**. Demographic characteristics of seroconverters and non-seroconverters.

| **Variable** | | **Seroconverters**  N=143 | **Non-seroconverters**  N=534 | **P value** |
| --- | --- | --- | --- | --- |
| Country | South Africa | 55 (38.5%) | 217 (40.6%) | 0.64 |
|  | Zambia | 88 (61.5%) | 317 (59.4%) |  |
| Age | 18-24 years | 63 (44.1%) | 88 (16.5%) | **<0.001** |
|  | 25+ years | 80 (55.9%) | 446 (83.5%) |  |
| Sex | Male | 24 (16.8%) | 104 (19.5%) | 0.47 |
|  | Female | 119 (83.2%) | 430 (80.5%) |  |

Legend for Table.

The table shows demographic characteristics of the 143 seroconverters and the 534 non-seroconverters who had HIV genotyping results.

**Supplemental File 5.** Antiretroviral drug testing.

Samples were tested using a qualitative assay that detects the following antiretroviral (ARV) drugs: non-nucleoside reverse transcriptase inhibitors (NNRTIs): efavirenz (EFV), nevirapine (NVP), and rilpivirine; nucleoside/nucleotide reverse transcriptase inhibitors (NRTIs): abacavir (ABC), emtricitabine (FTC), lamivudine (3TC), stavudine, tenofovir (TFV), and zidovudine (ZDV); protease inhibitors (PIs): amprenavir, atazanavir, darunavir, indinavir, lopinavir (LPV), nelfinavir, ritonavir (r), saquinavir, tipranavir; integrase strand transfer inhibitors (INSTIs): dolutegravir, elvitegravir, and raltegravir; CCR5-antagonist: maraviroc. The lower limit of detection for the assay is 20 ng/mL with the following exceptions: 5 ng/mL for ABC; 50 ng/mL for APV, DTG, EVG, NFV, and RAL; 100 ng/mL for TPV, and 150 ng/mL for EFV. The tables show results from ARV testing in different participant groups, by country and overall.

1. ARV drugs detected in seroconverters

Samples were available for ARV drug testing for 139 of the 143 seroconverters who had HIV genotyping results. ARV drugs were detected in samples from two of those participants.

| **ARV drug classes** | **ARV drugs** | **South Africa** | **Zambia** | **Total** |
| --- | --- | --- | --- | --- |
| 1 NNRTI + 2 NRTIs | EFV + 3TC + TFV | 0 | 2 | 2* |

* One participant reported current antiretroviral treatment (ART) and one reported having received ART for prevention of mother-to-child transmission of HIV (PMTCT).

1. ARV drugs detected in non-seroconverters

Samples were available for ARV drug testing for all 534 non-seroconverters who had HIV genotyping results. ARV drugs were detected in samples from 94 of those participants.

| **ARV drug classes** | **ARV drugs** | **South Africa** | **Zambia** | **Total** |
| --- | --- | --- | --- | --- |
| **1 NNRTI + ≥1 NRTIs** | EFV + 3TC + TFV | 1 | 46 | 47 |
|  | EFV + FTC + TFV | 15 | 0 | 15 |
|  | EFV + ABC + 3TC | 0 | 1 | 1 |
|  | EFV + 3TC | 0 | 1 | 1 |
|  | EFV + FTC | 2 | 0 | 2 |
|  | EFV + 3TC + FTC + TFV | 0 | 1 | 1 |
|  | EFV + TFV | 1 | 0 | 1 |
|  | NVP + 3TC | 0 | 2 | 2 |
|  | NVP + 3TC + TFV | 0 | 2 | 2 |
|  | Total |  |  | 72 |
| **1 NNRTI** | EFV | 3 | 8 | 11 |
|  | NVP | 1 | 3 | 4 |
|  | Total |  |  | 15 |
| **1 PI(r) + ≥1 NRTIs** | LPV/r + 3TC | 2 | 0 | 2 |
|  | LPV + 3TC | 0 | 1 | 1 |
|  | LPV/r + 3TC + ZDV | 1 | 0 | 1 |
|  | Total |  |  | 4 |
| **1 PI** | LPV | 1 | 0 | 1 |
| **1 NRTI** | 3TC | 1 | 0 | 1 |
| **2 NNRTIs + 2 NRTIs** | EFV + NVP + 3TC + TFV | 0 | 1 | 1 |
| **TOTAL** |  | **28** | **66** | **94*** |

* Sixty-nine (73.4%) of the 94 participants reported prior or current ART (64 reported current ART and five reported having received ART for PMTCT.

**Supplemental File 6.** Characteristics of participants with transmitted drug resistance.

The table show characteristics of seroconverters and non-seroconverters who were classified as antiretroviral (ARV) drug naïve and had HIV mutations detected that are used for surveillance of transmitted drug resistance (Bennett DE, et al. Drug resistance mutations for surveillance of transmitted HIV-1 drug-resistance: 2009 update. PLoS One. 2009;4(3):e4724). Fourteen seroconverters and 49 non-seroconverters were classified as having transmitted drug resistance.

**A. Transmitted drug resistance in seroconverters**

| **#** | **Study arm** | **Country** | **Sex** | **Age**  **(years)** | **TDR surveillance mutations** | | |
| --- | --- | --- | --- | --- | --- | --- | --- |
|  |  |  |  |  | **NNRTI** | **NRTI** | **PI** |
| 1 | A | South Africa | Male | 25+ | K103N |  |  |
| 2 | A | South Africa | Female | 25+ | K103N |  |  |
| 3 | A | South Africa | Female | 25+ |  |  | M46M/I |
| 4 | A | South Africa | Female | 25+ |  |  | M46M/L |
| 5 | A | South Africa | Female | 18-24 | V106M |  |  |
| 6 | A | Zambia | Female | 25+ | G190A |  |  |
| 7 | A | Zambia | Female | 25+ | K103N |  |  |
| 8 | A | Zambia | Male | 18-24 | K103N |  |  |
| 9 | A | Zambia | Female | 25+ |  | K219R |  |
| 10 | A | Zambia | Male | 18-24 | Y181C |  |  |
| 11 | B | Zambia | Female | 25+ | V106M, G190A | M41L, L74I, V75S, T215C/Y |  |
| 12 | C | South Africa | Male | 25+ | K103N |  |  |
| 13 | C | South Africa | Female | 25+ | V106M |  |  |
| 14 | C | Zambia | Female | 25+ |  | D67G |  |

**B. Transmitted drug resistance in non-seroconverters**

| # | **Study Arm** | **Country** | **Sex** | **Age**  **(years)** | **TDR surveillance mutations** | | |
| --- | --- | --- | --- | --- | --- | --- | --- |
|  |  |  |  |  | **NNRTI** | **NRTI** | **PI** |
| 1 | A | South Africa | Female | 25+ | K101E, G190A |  |  |
| 2 | A | South Africa | Female | 25+ | K103K/N |  |  |
| 3 | A | South Africa | Female | 25+ | K103K/N/S |  |  |
| 4 | A | South Africa | Female | 25+ | K103K/N |  | M46L |
| 5 | A | South Africa | Female | 25+ | K103N |  |  |
| 6 | A | South Africa | Male | 25+ | K103N |  |  |
| 7 | A | South Africa | Female | 18-24 | K103N |  |  |
| 8 | A | South Africa | Male | 25+ | K103N |  |  |
| 9 | A | South Africa | Female | 25+ | K103N | M184M/V |  |
| 10 | A | South Africa | Female | 18-24 |  | K219Q |  |
| 11 | A | South Africa | Female | 25+ | V106M | K65K/R, M184M/I/V |  |
| 12 | A | South Africa | Female | 18-24 | Y181C |  |  |
| 13 | A | Zambia | Female | 25+ | K103K/N |  |  |
| 14 | A | Zambia | Female | 25+ | K103K/N |  |  |
| 15 | A | Zambia | Female | 25+ | K103N |  |  |
| 16 | A | Zambia | Male | 25+ | K103N |  |  |
| 17 | A | Zambia | Female | 25+ | V106M |  |  |
| 18 | A | Zambia | Female | 18-24 |  |  | M46L |
| 19 | A | Zambia | Female | 25+ |  |  | M46M/L |
| 20 | B | South Africa | Female | 25+ | K103K/N |  |  |
| 21 | B | South Africa | Female | 25+ | K103N |  |  |
| 22 | B | South Africa | Female | 25+ | K103N |  |  |
| 23 | B | South Africa | Female | 25+ | K103N |  |  |
| 24 | B | South Africa | Female | 25+ | K103N |  | I54I/T |
| 25 | B | South Africa | Female | 25+ | V106M |  |  |
| 26 | B | South Africa | Female | 25+ | K103K/N |  |  |
| 27 | B | Zambia | Female | 25+ | K101K/E |  |  |
| 28 | B | Zambia | Female | 18-24 | K101K/E |  |  |
| 29 | B | Zambia | Female | 25+ | K103N |  |  |
| 30 | B | Zambia | Female | 25+ |  |  | I54I/S |
| 31 | B | Zambia | Female | 25+ | V106V/M |  |  |
| 32 | B | Zambia | Female | 25+ |  |  | M46L |
| 33 | B | Zambia | Female | 25+ | K103N |  |  |
| 34 | C | South Africa | Female | 25+ | K101E |  |  |
| 35 | C | South Africa | Female | 18-24 | K103K/N |  |  |
| 36 | C | South Africa | Male | 25+ | K103K/N |  |  |
| 37 | C | South Africa | Female | 25+ | K103K/N, V106V/M |  |  |
| 38 | C | South Africa | Female | 25+ | K103N |  |  |
| 39 | C | South Africa | Female | 18-24 | K103N |  |  |
| 40 | C | South Africa | Male | 25+ | K103N |  |  |
| 41 | C | South Africa | Female | 25+ | K103N, P225H |  |  |
| 42 | C | South Africa | Male | 25+ | V106M |  |  |
| 43 | C | Zambia | Female | 18-24 | G190G/E |  |  |
| 44 | C | Zambia | Male | 18-24 | K101E |  |  |
| 45 | C | Zambia | Female | 18-24 | K103N |  |  |
| 46 | C | Zambia | Female | 25+ |  |  | I85I/V |
| 47 | C | Zambia | Female | 25+ |  |  | G73G/S |
| 48 | C | Zambia | Female | 25+ |  |  | I85I/V |
| 49 | C | Zambia | Female | 25+ |  |  | I85V |

Abbreviations: TDR: transmitted drug resistance; NNRTI: non-nucleoside reverse transcriptase inhibitor, NRTI: nucleoside/nucleotide reverse transcriptase inhibitor; PI: protease inhibitor.
